# Peroxisome Proliferator-Activated Receptors: “Key” Regulators of Neuroinflammation after Traumatic Brain Injury

**DOI:** 10.1155/2008/538141

**Published:** 2008-03-27

**Authors:** Philip F. Stahel, Wade R. Smith, Jay Bruchis, Craig H. Rabb

**Affiliations:** ^1^Department of Orthopedic Surgery, Denver Health Medical Center, School of Medicine, University of Colorado, 777 Bannock Street, Denver, CO 80204, USA; ^2^Division of Neurosurgery, Department of Surgery, Denver Health Medical Center, School of Medicine, University of Colorado, 777 Bannock Street, Denver, CO 80204, USA

## Abstract

Traumatic brain injury is characterized by neuroinflammatory pathological sequelae which contribute to brain edema and delayed neuronal cell death. Until present, no specific pharmacological compound has been found, which attenuates these pathophysiological events and improves the outcome after head injury. Recent experimental studies suggest that targeting peroxisome proliferator-activated receptors (PPARs) may represent a new anti-inflammatory therapeutic concept for traumatic brain injury. PPARs are “key” transcription factors which inhibit NF*κ*B activity and downstream transcription products, such as proinflammatory and proapoptotic cytokines. The present review outlines our current understanding of PPAR-mediated neuroprotective mechanisms in the injured brain and discusses potential future anti-inflammatory strategies for head-injured patients, with an emphasis on the putative beneficial combination therapy of synthetic cannabinoids (e.g., dexanabinol) with PPAR*α* agonists (e.g., fenofibrate).

## 1. INTRODUCTION

Research efforts in recent years have provided increasing evidence that the intracerebral inflammatory response is in large part responsible for the devastating neuropathological sequelae and poor outcome of traumatic brain injury [1–3]. The extent of brain damage is determined by primary and secondary injury patterns. While the primary injury results from mechanical forces applied to the skull and brain at the time of impact, secondary brain injury occurs as a delayed consequence of trauma [4–7]. Secondary brain injuries are mediated by endogenous pathophysiological processes which lead to an overwhelming neuroinflammation in the injured brain [6, 8–10]. The main risk factors for developing secondary brain injuries are hypoxemia and systemic hypotension which occur frequently in the trauma patient [11, 12]. These conditions contribute to the ischemic brain damage and perpetuate the intracerebral inflammatory reaction through ischemia/reperfusion-mediated mechanisms [13]. Peroxisome proliferator-activated receptors (PPARs) are ligand-activated transcription factors of the nuclear receptor superfamily which have recently been shown to exert anti-inflammatory properties in acute neurological disorders. These include cerebrovascular stroke, intracerebral hemorrhage, spinal cord injury, and traumatic brain injury [14–21]. The present paper provides an overview on the so far known anti-inflammatory properties of PPARs in brain injury and discusses potential pharmacological properties of PPAR agonists as future neuroprotective agents.

## 2. BIOLOGICAL FUNCTIONS OF PPARS

**Figure 1:**
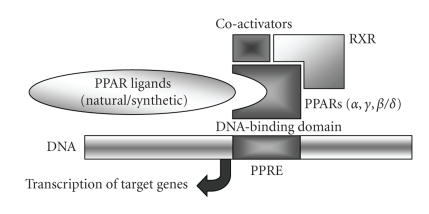
*Mechanism of gene transcription through ligand binding on peroxisome proliferator-activated receptors (PPARs)*. In presence of coactivating stimuli, PPARs heterodimerize with retinoid X receptors (RXR) to form active transcription factors. The DNA binding domain on PPAR-RXR heterodimers induces the transcription of target genes by binding to peroxisome proliferator-response elements (PPRE's) which consist of DNA-specific sequences.

PPARs are nuclear membrane-associated transcription factors belonging to the nuclear receptor family [22]. Three isotypes with a differential tissue distribution have been described: PPAR*α* (NR1C1), PPAR*β*/*δ* (NR1C2), and PPAR*γ* (NR1C3) [23, 24]. While PPAR*β*/*δ* has an ubiquitous expression, PPAR*α* and PPAR*γ* are mainly expressed in tissues with high fatty acid catabolism, such as adipose tissue, liver, kidney, and skeletal muscle [25]. Mechanistically, PPARs are activated by heterodimerization with the retinoid-X receptor (RXR) into biologically active transcription factors. PPAR-RXR heterodimers induce the transcription of candidate genes by binding to so-called peroxisome proliferator-response elements (PPRE's) consisting of DNA-specific sequences (see Figure 1).

PPARs exert a wide variety of physiological functions [24, 26]. They play a central role in the regulation of lipid and lipoprotein metabolism and glucose homeostasis, and have been shown to mediate cellular proliferation and programmed cell death (apoptosis) [27–31]. PPARs have furthermore been involved in bone metabolism and in pathologies of the cardiovascular system and the lung [32–35]. PPAR*α* has been attributed important immunological functions due to its expression on monocytes/macrophages, T cells, and vascular endothelial cells. PPAR*γ* appears to play a crucial role in the regulation of proliferation and differentiation of various cell types. While the biological role of PPAR*β*/*δ* has not been defined in detail, recent data imply an antiapoptotic and anti-inflammatory effect after tissue injury, both in vitro and in vivo [29].

From an immunological viewpoint, PPARs have been identified as important regulators of inflammatory gene expression [36–40]. PPARs have also been shown to attenuate adaptive immune responses by inhibiting helper T cell functions and by mediating apoptosis of B cells [41, 42]. PPARs are activated by naturally ocurring fatty acid derivatives, eicosanoides, and by synthetic pharmacological agents, such as fibrates (PPAR*α*) and glitazones (PPAR*γ*) [18, 22, 43]. PPAR ligands have been shown to exert anti-inflammatory activities in various cell types by inhibiting the gene expression for proinflammatory cytokines, metalloproteinases, and hepatic acute-phase proteins.

## 3. PPARS: “KEY” REGULATORS OF NEUROINFLAMMATION

Mechanistically, the activation of PPAR*α* has been shown to inhibit proinflammatory gene transcription by repressing the central inflammatory transcription factor, nuclear factor-*κ*B (NF-*κ*B) [43–45]. Along with suppression of NF-*κ*B, PPAR*α* acts by inhibition of signal transduction through activator protein-1 (AP-1) signaling [43]. It appears that the inhibitory effect of PPAR*α* on these crucial inflammatory transcription factors creates a negative feedback loop for controlling acute posttraumatic inflammation [44–46]. First in vivo data on the involvement of PPARs in the regulation of inflammation were reported from studies in PPAR*α* knockout mice [47]. Cuzzocrea et al. showed that the targeted deletion of the PPAR*α* gene leads to a significantly increased inflammatory response in different experimental models of acute inflammation outside the central nervous system (CNS) [47]. Within the CNS, the constitutive expression of PPARs has been described for some time [48, 49]. Interestingly, PPAR gene expression was detected not only on vascular endothelial cells in the brain and spinal cord, but also on resident cells in the CNS, such as neurons and glial cells [49].

## 4. ROLE OF PPARS IN CNS INJURY

In recent years, experimental studies in models of cerebral ischemia/reperfusion injury, ischemic stroke, intracerebral hemorrhage, and spinal cord injury have revealed a crucial role of PPARs in attenuating neuroinflammation and neuronal cell death in the injured CNS (see Table 1) [14–16, 19, 20, 50–52]. PPAR*α* gene-deficient mice (PPAR*α*−/−) were shown to have a significantly worsened neurological outcome, associated with an increased neuroinflammatory response to experimental spinal cord injury, as compared to wild-type littermates [16]. The postulated neuroprotective effects of natural PPAR*α* ligands include the attenuation of polymorphonuclear leukocyte (PMNL) recruitment and associated neurotoxicity, as determined by a significantly reduced expression of myeloperoxidase in the injured spinal cord of PPAR*α*−/− mice [16]. In addition, tumor necrosis factor (TNF), a “key” mediator of neuroinflammation and neurotoxicity, was shown to be upregulated and associated with neuronal apoptosis in the injured spinal cord of PPAR*α*−/− mice [16]. In traumatic brain injury, experimental studies in the past decade have shown that TNF is upregulated in the intracranial compartment within a few hours after trauma, and contributes to secondary neuronal injury [53–55]. The deleterious neurotoxic effects were shown to be abrogated by pharmacological inhibition of TNF [56]. Since PPARs inhibit proinflammatory gene transcription by attenuating NF-*κ*B signaling [43–45], the potent PPAR-mediated neuroprotective effects may be dependent on inhibition of NF-*κ*B-dependent proinflammatory cytokines released in the injured brain, such as TNF, interleukin (IL)-1, IL-8, IL-12, and IL-18 [57–61]. The central role of NF-*κ*B signaling in inflammation and oxidative stress explains why PPARs have been considered possible targets for neuroprotection in inflammatory CNS diseases, including traumatic brain injury [14, 20, 62, 63].

## 5. PHARMACOLOGY OF HEAD INJURY: ARE PPAR-AGONISTS AND CANNABINOIDS THE LONG SOUGHT “GOLDEN BULLET”?

A wide variety of natural and synthetic PPAR*γ* agonists have been described in recent years as regulators of microglial activation and cerebral inflammation [63]. For example, the thiazolinedione pioglitazone has been shown to reduce the extent of neuroinflammation and the severity of disease in experimental autoimmune encephalomyelitis (EAE), the animal model for multiple sclerosis (MS) [64, 65]. A recent case report described the impressive clinical improvement of a patient with chronic progressive MS, after a 3-year period of treatment with pioglitazone [66]. This unexpected clinical recovery implies that PPAR*γ* agonists may represent a promising new strategy for attenuating neuroinflammation in patients with CNS autoimmune diseases [62, 63, 67].

In cerebrovascular stroke, the combination therapy of a PPAR*γ* agonist (rosiglitazone) with an antiexcitotoxic glutamate receptor antagonist (MK-801) led to an improved neurological recovery in rats undergoing middle cerebral artery occlusion [18]. A study by another group assessed the therapeutic efficacy of two different PPAR*γ* agonists, rosiglitazone and pioglitazone, in a rat model of cerebral ischemia/reperfusion injury [50]. The authors showed that the pretreatment with either compound led to a significant attenuation of inflammation and oxidative stress in injured rat brains [50].

Pharmacological ligands to PPAR*α*, such as fenofibrate, have also been shown to exert neuroprotective effects in inflammatory CNS conditions. Deplanque et al. demonstrated a significant neuroprotective effect of fenofibrate administration in C57BL/6 mice with cerebrovascular stroke [68]. The authors suggested that PPAR*α* may represent a new pharmacological target to reduce the neuroinflammatory and neuropathological sequelae of cerebrovascular stroke [68].

In traumatic brain injury, the PPAR*α* agonist fenofibrate appears to represent a highly promising new anti-inflammatory compound. Besson et al. assessed the pharmacological role of fenofibrate in a model of experimental fluid-percussion injury in adult male Sprague-Dawley rats [21]. The authors revealed that the administration of fenofibrate during a clinically relevant therapeutic “time window of opportunity” at 1 hour after trauma mediated a significant posttraumatic neuroprotection. This was demonstrated by improved neurological scores in the fenofibrate group at 24 hours and 7 days after trauma, compared to vehicle-treated animals [21]. Morphologically, fenofibrate treatment resulted in significantly decreased extent of brain edema at 24 hours after head injury, compared to the placebo group. The authors furthermore described a marked reduction in intercellular adhesion molecule (ICAM)-1 expression at the protein level by immunohistochemistry in injured rat brain sections after fenofibrate administration [21]. This finding implies a reduced extent of intracerebral immunoactivation and neuroinflammation in rats treated by the PPAR*α* agonist, compared to vehicle controls.

**Figure 2:**
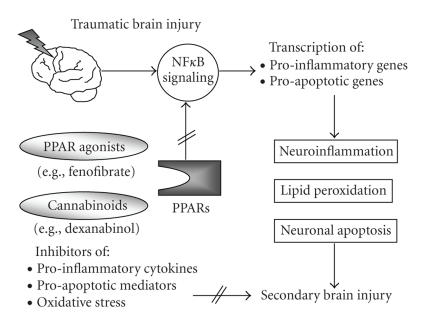
* Working hypothesis of PPAR-mediated mechanisms of neuroprotection after traumatic brain injury*. The neuropathological sequelae of head injury include the posttraumatic activation of NF*κ*B-dependent inflammatory genes. The transcription of neuroinflammatory mediators in the injured brain induces and perpetuates the intracranial inflammatory response and leads to formation of brain edema and adverse outcome. Activation of PPARs by binding of synthetic ligands, such as the PPAR*α* agonist fenofibrate, leads to inhibition of NF*κ*B and of downstream transcribed proinflammatory and proapoptotic mediators. In addition, cannabinoids have a dual neuroprotective function, (1) by acting as ligands to PPARs and (2) by inhibiting “key” mediators of neuroinflammation and apoptosis, such as tumor necrosis factor (TNF). The combination therapy of synthetic PPAR agonists and cannabinoids may represent the long sought pharmacological “golden bullet” for the treatment of traumatic brain injury in the future.

A more recent follow-up study by the same research group assessed the role of PPAR*α* in modulating the oxidative stress in the injured rat brain [17]. Oxidative stress and ischemia/reperfusion-mediated injuries contribute significantly to the extent of posttraumatic intracerebral inflammation and delayed secondary brain damage after head injury [13, 69, 70]. Pathophysiologically, contused brain areas are surrounded by a penumbra zone which is hypoperfused due to traumatic vascular damage, loss of cerebrovascular autoregulation, and systemic hypotension. After resuscitation, the hypoperfused, ischemic brain areas in the penumbra zone are reperfused, which leads to activation of the complement cascade and of reactive oxygen intermediates by activation of the xanthine oxidase [71, 72]. Oxygen-derived free radicals such as hydroxyl ions, hydrogen peroxide, and superoxide anion induce lipid peroxidation, cell membrane disintegration, and delayed neuronal cell death (see Figure 2). Lipid peroxidation is facilitated in the brain due to its genuine vulnerability to oxidative stress based on specific morphological characteristics, such as a high ratio of “membrane to cytoplasm” and high levels of polyunsaturated fatty acids in the CNS [70]. In addition to reactive oxygen intermediates, the generation of nitric oxide (NO) by inducible NO synthase (iNOS) up-regulation also occurs after head injury and adds to the extent of secondary brain damage [73]. Metabolites emerging from the interaction between superoxide anion and NO, such as the highly reactive oxidant peroxynitrite, have been shown to mediate neurotoxicity and delayed neuronal cell death after traumatic brain injury [74].

The pharmacological administration of the PPAR*α* agonist fenofibrate after experimental fluid-percussion injury resulted in a significant decrease of intracerebral iNOS expression [17]. This was associated with a decreased neuroinflammation in the injured brain and an improved neurological recovery after trauma [17]. These important findings imply that the attenuation of oxidative stress may represent a “key” mechanistic aspect of PPAR-mediated neuroprotection after head injury. The pleiotropic beneficial effects of PPARs in the injured brain, however, are far from being elucidated in detail until present. For example, in contrast to PPAR*α*, no studies have yet been performed to analyze the effect of PPAR*γ* in experimental models of traumatic brain injury (see Table 1).

Despite increasing insights into the pathophysiological mechanisms of posttraumatic neuroinflammation and neurodegeneration, clinical neuroprotection trials have failed to provide a benefit of anti-inflammatory pharmacological strategies with regard to the outcome after head injury [75, 76]. Cannabinoids have recently evolved as a promising new therapeutic avenue for neuroprotection after head injury [77–79]. This group of compounds consists of natural (endocannabinoids) and synthetic ligands, such as dexanabinol (HU-211). The endocannabinoid 2-arachidonoyl glycerol (2-AG) has received increased attention in recent years due to its strong neuroprotective effect after head injury, by inhibition of proinflammatory cytokines, reactive oxygen intermediates, and excitotoxic aminoacids, such as glutamate [80, 81]. The pharmacological agent dexanabinol was shown to mediate neuroprotection by inhibition of TNF production in injured rodent brains [77, 82] and was recently proposed as an effective neuroprotective strategy to reduce the extent of secondary brain injury in humans (see Figure 2) [78, 79]. Dexanabinol (HU-211) is a nonpsychotropic, synthetic cannabinoid which exerts beneficial effects by cytokine inhibition and radical scavenging associated with reduction of brain edema [77–79, 82]. Cannabinoids were attributed a new role as neuroprotective agents by agonistic action to PPARs [83]. The functional interaction between cannabinoids and PPARs was first described based on the finding of oleylethanolamide (OEA), a lipid derivate structurally related to anandamide, as a regulator of feeding behavior in rats through activation of PPAR*α* [68, 84]. Aside from OEA, which is a low-affinity agonist to cannabinoid receptors, other cannabinoids were recently described as PPAR ligands [83]. As such, Δ^9^-tetrahydrocannabinol (THC) was found to activate PPAR*γ* in human cell lines [85]. Of particular interest for neuroprotection in traumatic brain injury is the finding that the potent endocannabinoid 2-AG [80, 81] has been found to suppress the proinflammatory cytokine IL-2 through PPAR*γ* signaling, independent of 2-AG binding to cannabinoid receptors [86]. Future studies will have to determine whether cannabinoids represent the long sought “golden bullet” for reduction of secondary brain damage after head injury. It seems reasonable to suggest that a combination of neuroprotective cannabinoids, such as dexanabinol, with other potent anti-inflammatory therapeutic agents, such as synthetic PPAR ligands, may represent a promising new therapeutic avenue for improving the outcome of traumatic brain injury.

## Figures and Tables

**Table 1:** Selected publications on the role of PPARs in CNS injury and inflammation.

Models of CNS injury and neuroinflammation	PPAR isotype	Main findings	Reference no.
Different models of CNS injury	PPAR*γ*	Review on the mechanisms of neuroprotection by PPAR*γ* agonists	Kapadia et al. [20]
Different models of CNS injury	PPAR*α*, PPAR*γ*	Review on pharmacological neuroprotection by PPARs	Bordet et al. [14]
Brain inflammation	PPAR*γ*	Review on regulation of microglial activation by PPAR*γ* agonists	Bernardo and Minghetti [63]
Spinal cord injury	All isotypes	Review on the role of PPAR signal transduction in spinal cord injury	Van Neerven and Mey [15]
Spinal cord injury	PPAR*α*	Experimental model of spinal cord injury in PPAR*α* gene knockout mice. Lack of PPAR*α* leads to worse outcome and increased neuroinflammation.	Genovese et al. [16]
Cerebral ischemia/reperfusion injury	PPAR*γ*	The PPAR*γ* agonists rosiglitazone and pioglitazone exert neuroprotective effects in a rat model of cerebral ischemia/reperfusion injury by reducing neuroinflammation and oxidative stress.	Collino et al. [50]
Intracerebral hemorrhage	PPAR*γ*	PPAR*γ* expressed by microglia and macrophages promotes the resolution of intracerebral hemorrhage and attenuates the neuroinflammatory response.	Zhao et al. [19]
Traumatic brain injury	PPAR*α*	The PPAR*α* agonist fenofibrate reduces brain edema and improves the neurological outcome after experimental fluid percussion brain injury in male Sprague-Dawley rats.	Besson et al. [21]
Traumatic brain injury	PPAR*α*	The PPAR*α* agonist fenofibrate promotes neurological recovery by reducing inflammation and oxidative stress in rat brains after experimental fluid percussion brain injury.	Chen et al. [17]
Neuroinflammation	All isotypes	Review on the interaction between cannabinoids and PPARs as inhibitors of neuroinflammation	Sun and Bennett [83]
